# Revealing the Effect of α’ Decomposition on Microstructure Evolution and Mechanical Properties in Ti80 Alloy

**DOI:** 10.3390/ma17102238

**Published:** 2024-05-09

**Authors:** Chunhong Xiao, Bin Hu, Jinyang Ge, Bin Kong, Deng Luo, Xiaoyong Zhang, Kechao Zhou

**Affiliations:** 1 State Key Laboratory of Powder Metallurgy, Materials Science and Engineering, Powder Metallurgy Research Institute, Central South University, Changsha 410083, China; xch1320613602@163.com (C.X.); 213301053@csu.edu.cn (B.H.); 203301071@csu.edu.cn (J.G.); zhoukechao@csu.edu.cn (K.Z.); 2 Hunan Xiangtou Goldsky Titanium Metal Co., Ltd., No. 116, Linyu Road, Yuelu Zone, Changsha 410221, China; 633kb@163.com; 3 Xiangtan Iron & Steel Group Co., Ltd., Yue Tang District, Xiangtan 411104, China; 213311053@csu.edu.cn

**Keywords:** Ti80, solution treatment and aging, α’ martensitic decomposition, nano-β, strength–plasticity matching

## Abstract

Three types of solution treatment and aging were designed to reveal the α’ decomposition and its effect on the mechanical properties of near-α Ti-80 alloy, as follows: solution at 970 °C then quenching (ST), ST + aging at 600 °C for 5 h (STA-1), and ST + aging 600 °C for 24 h (STA-2). The results show that the microstructures of the ST samples were mainly composed of equiaxed α_p_ and acicular α’, with a large number of dislocations confirmed by the KAM results. After subsequent aging for 5 h, α’ decomposed into acicular fine α_s_ and nano-β (intergranular β, intragranular β) in the STA-1 specimen, which obstructed dislocation motion during deformation, resulting in the STA-1 specimen exhibiting the most excellent yield strength (1012 MPa) and maintaining sufficient elongation (8.1%) compared with the ST (898 MPa) and STA-2 (871 MPa) samples. By further extending the aging time to 24 h, the size of acicular α_s_ and nano-β gradually increased while the density of dislocations decreased, which resulted in a decrease in strength and an increase in plasticity. Based on this, a microstructures–properties correlation model was proposed. This study provides a new method for strength–plasticity matching of near-α titanium alloys through α’ decomposition to acicular α_s_+nano-β.

## 1. Introduction

A near-α titanium alloy, Ti-6Al-3Nb-2Zr-1Mo (Ti80), is attractive for marine applications due to its high specific strength, excellent impact toughness, and good seawater corrosion resistance [1,2]. However, with the development of marine engineering, higher requirements are proposed for the mechanical properties of the alloy, including better strength and plasticity. In addition to thermomechanical processing, heat treatment is a common way to obtain the desired properties by adjusting the microstructure of titanium alloys through controlling the phase transition [3,4]. Generally, extremely fine α_s_ has a greater capacity to obstruct dislocation motion, thereby increasing the strength of the alloy, and equiaxed primary α_p_ can improve the plasticity of the alloy [5,6].

Solution treatment and aging is a common heat treatment to strengthen titanium alloys [7,8]. For titanium alloys with relatively low content of β- stable elements, after solution treatment in the β-phase region and quenching, the β phase undergoes β → α′ displacive martensitic transformation, resulting in disorderly distributed formation of a fully acicular α′ microstructure [9,10]. The displacive transformation is associated with the generation of a large number of dislocations [11], which results in poor plasticity [12,13]. Even after subsequent aging, it is challenging to achieve a substantial increase in plasticity. Su et al. [14] confirmed this view in the Ti-6Al-4V alloy, where solution-treated samples showed a poor elongation of ~3%, and the elongation increased slightly to ~6% with the subsequent aging process. Similar results were reported in Ti-30Zr-5Al-2.5Sn alloys [15] and in TA19 [10].

To overcome the lack of plasticity caused by fully acicular α′ microstructure after solution treatment in the β phase region and aging, solution treatment within the α+β phase region and aging have been investigated [16,17]. Solution treatment within the α+β phase region can retain a certain amount of equiaxial primary α_p_. As a plastic phase, equiaxed α_p_ is capable of withstanding more plastic deformation during deformation, reducing stress concentrations and thus avoiding the generation of voids [18]. For instance, Matsumoto et al. [19] found that the alloy possessing both the primary equiaxial α_p_ phase and the acicular α′ phase exhibited better cold-rolling reduction (43.3%) than the full acicular α′-phase (24.6%) on a Ti-6Al-4V alloy. Zhu et al. [20] conducted solution and aging treatment in the α+β region on as-rolled Ti-6Al-4V, obtaining the equiaxial α_p_ phase and the β_t_. The tensile strength of the alloy had increased from 1051 MPa in the initial state to 1254 MPa, and retained the elongation with 10.4%. Chen et al. [21] investigated the influence on the tensile properties of Ti-6121 alloys after solution treatment in the α+β phase region and aging. The strength increased from 1096 MPa in the rolled state to 1387 MPa, and the elongation reached 11%. Similar results were also reported by Mandal et al. [22] in a α+β titanium alloy and Shi et al. [23] in a near-α titanium alloy. For α, near-α, and α+β titanium alloys with relatively low contents of β-stable elements, as mentioned above, solution treatment in the α+β phase region and aging have been widely used to modulate the microstructures and optimize their properties, and have achieved good outcomes. However, as for the solution treatment and aging of near-α titanium alloys, the evolution of the martensitic α′ phase and its effect on mechanical properties require further investigation, and strengthening mechanisms and models need to be further developed.

In this work, the microstructure characteristics and mechanical properties of Ti80 alloy subjected to solution treatment within the α+β phase region and subsequent aging were comparatively investigated. Through optical microscope (OM), scanning electron microscope (SEM), electron backscattered diffraction (EBSD), and transmission electron microscope (TEM) characterization, the microstructural evolution, including the decomposition mechanisms of the α′ phase, the change in dislocation density, and their effects on mechanical properties, were revealed. Furthermore, a microstructures–properties correlation model was constructed. These findings may provide a new concept for strength–plasticity matching of near-α titanium alloys by α’ decomposing to acicular α_s_+nano-β.

## 2. Experimental Procedures

**Figure 1:**
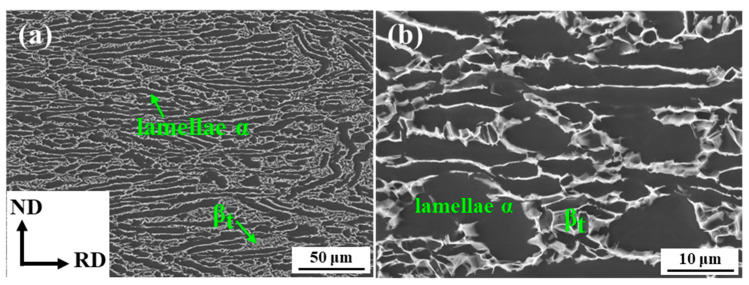
Initial microstructure of Ti80 alloy: (**a**) low magnification initial microstructure of Ti80 alloy; (**b**) high magnification initial microstructure of Ti80 alloy.

In this study, an as-rolled Ti80 alloy plate was provided by Hunan Xiangtou Goldsky Titanium Mental Co., Ltd. (Changsha, China), and its chemical composition is shown in Table 1. The α/β transformation temperature (T_β_) of this billet, measured using the metallographic method, was 1000 °C. Figure 1 illustrates the initial microstructure of the Ti80 plate, which was composed of lamellar α and β_t_ phases. The lamellar α phase was parallel to the rolling direction (RD).

**Figure 2:**
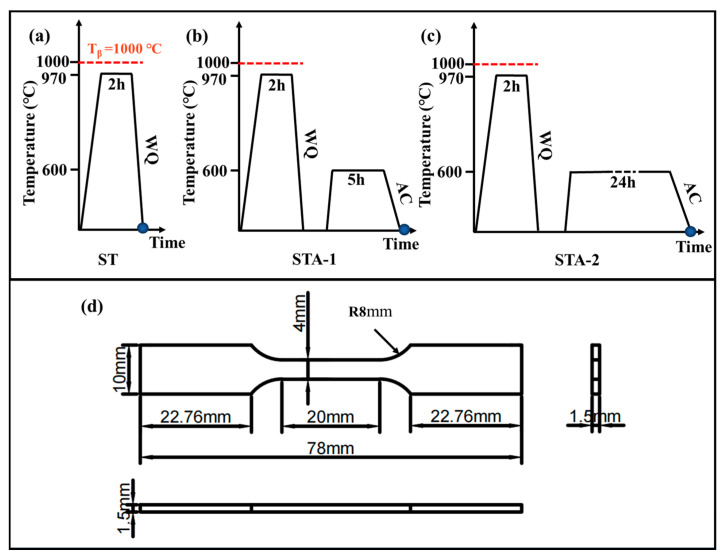
(**a**–**c**) Schematic diagram of heat treatment process: (**a**) ST; (**b**) STA-1; (**c**) STA-2. (**d**) Diagram of the tensile specimen.

The specimens with sizes of 80 mm × 22 mm × 6 mm were machined from the billet for heat treatment experiments. The schematic diagram of the heat treatment process is presented in Figure 2a–c. The three heat treatment methods are as follows: (I) solution at 970 °C for 2 h, followed by quenching (ST sample); (II) solution at 970 °C for 2 h, followed by quenching, aging at 600 °C for 5 h, and air cooling (STA-1 sample); and (III) solution at 970 °C for 2 h, followed by quenching, aging at 600 °C for 24 h, and air cooling (STA-2 sample). In this study, the alloy block was sealed within a vacuum quartz tube for the purpose of heat treatment in order to prevent the influence of oxygen contamination.

In terms of mechanical performance testing samples, a dog bone tensile specimen with a gauge size of 78 mm × 10 mm × 1.5 mm was excised from the heat-treated specimens, as shown in Figure 2d. The effect of wire cutting was eliminated after mechanical grinding of the tensile samples. Tensile tests were carried out using an Instron 3369 mechanical testing machine (Instron, Norwood, MA, USA), with a stretching speed of 2 mm/min. The displacement of the specimen was recorded using a YYJ-4/10-L extensometer (Suzhou Shenghui Precision Instrument Technology Co., Ltd., Suzhou, China). A tensile experiment with strains up to 3% was carried out using an IBTC-500 in situ mechanical test system (CARE Measurement and control, Tianjin, China), and the tensile speed was 0.002 mm/s. Upon reaching a strain of 3%, the tensile test was terminated. The strain of the ST, STA-1, and STA-2 samples was 3%, just passing the yield point.

Some small blocks were cut for the microstructure characterization; subsequently, this pieces were subjected to mechanical grinding with 240 #, 400 #, and 600 # metallographic sandpaper, followed by electrochemical polishing in Kroll’s solution (60% methanol, 35% butanol, and 5% perchloric acid) for 20 s. Scanning electron microscopy (SEM) was performed under a Tescan Mira4 electron microscope, (TESCAN, Brno, Czech Republic), and electron backscatter diffraction (EBSD) observation was conducted using a Regulus 8230 ultra-high-resolution large-beam cold field emission scanning electron microscope (Hitachi Limited, Tokyo, Japan). Thin films for transmission electron microscopy (TEM) were prepared by means of electrolytic polishing (Denmar Struers A/S, Copenhagen, Denmark) of Kroll’s solution at −23 °C using a double jet electrolytic apparatus, and TEM observations were conducted under F200X field emission transmission electron microscopy (TESCAN, Brno, Czech Republic). The size and the content of the α phase were statistically analyzed using Image J 1.54d software. The size and content of the α phase were quantified using Image J software.

## 3. Results

### 3.1. Mechanical Properties and Fracture Morphology

**Figure 3:**
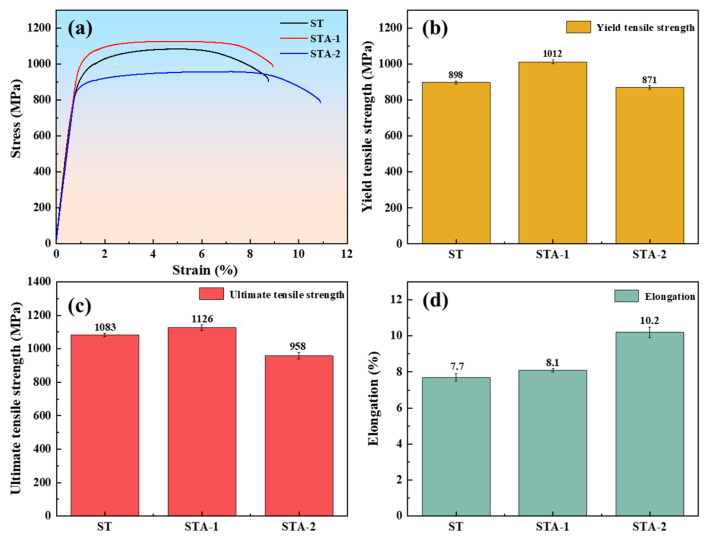
Tensile properties of Ti80 alloy subjected to three heat treatments: (**a**) engineering stress–strain curve; (**b**) ultimate tensile strength; (**c**) yield tensile strength; (**d**) elongation.

**Figure 4:**
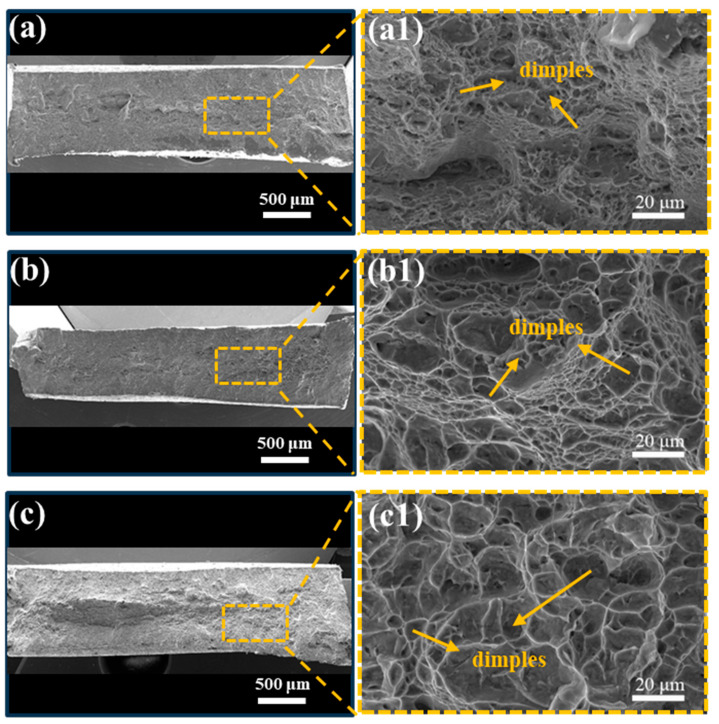
Fracture micrographs of different tensile test samples. (**a**,**a1**) ST, (**b**,**b1**) STA-1, and (**c**,**c1**) STA-2.

Figure 3 illustrates the engineering stress–strain curves and corresponding tensile properties of the Ti80 alloy subjected to three heat treatments. After solution at 970 °C for 2 h, followed by quenching, the yield tensile strength (YTS), ultimate tensile strength (UTS), and elongation of the ST specimen reached 898 MPa, 1083 MPa, and 7.7%, respectively. After aging at 600 °C for 5 h, the STA-1 specimen obtained improved strength and ductility, with the YTS, UTS, and elongation increasing to 1012 MPa, 1126 MPa, and 8.1%; the YTS was 10–20% higher than that already reported for Ti80 alloys (785 MPa~885 Ma) [3,4]. After extending the aging time to 24 h, the YTS of STA-2 decreased to 871 MPa, while the elongation increased to 10.2%. Figure 4 illustrates the fracture morphology of the Ti80 alloy tensile specimens subjected to three heat treatment conditions, respectively. Compared to those of ST and STA-1 samples, the dimples of the STA-2 specimen were significantly larger and deeper, which is consistent with the good plasticity of the STA-2 sample.

### 3.2. Microstructures Characteristics

**Figure 5:**
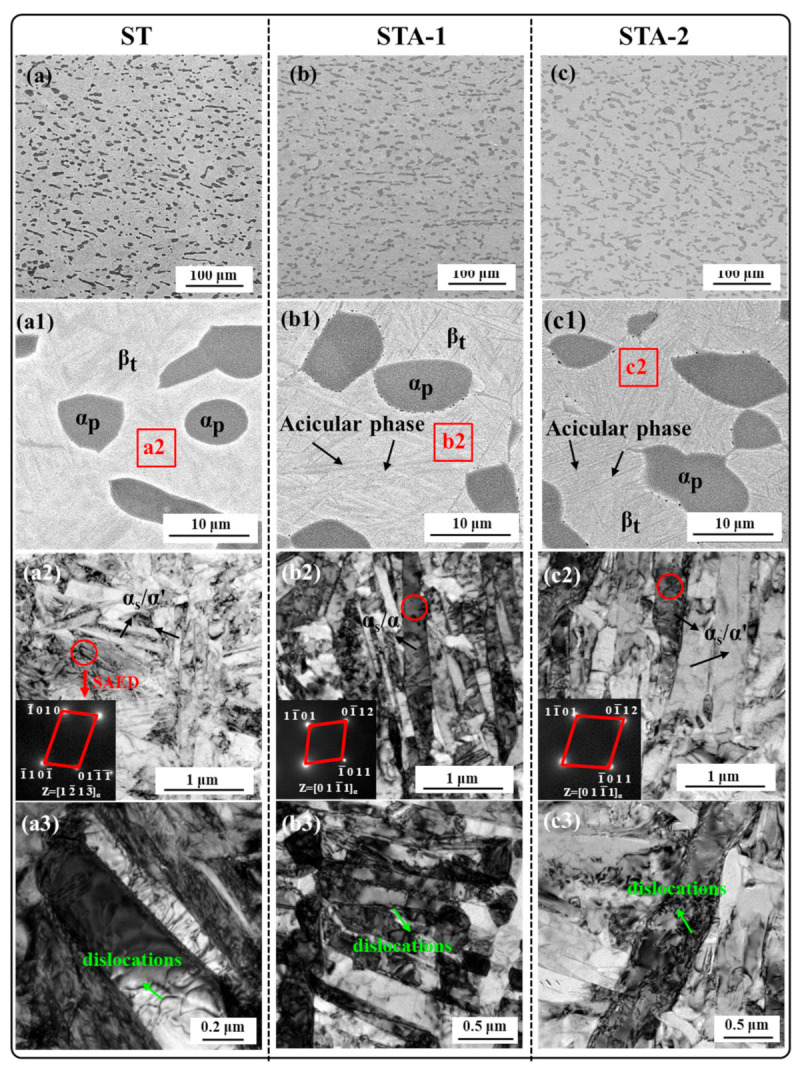
SEM and TEM microstructure and selected area electron diffraction pattern of the samples after heat treatment. (**a**–**c**) Low magnification SEM image; (**a1**–**c1**) High magnification SEM image; (**a2**–**c2**) TEM image corresponding to the red square in (**a1**–**c1**) and SAED pattern corresponding to the red circle; (**a3**–**c3**) High magnification TEM image.

The microstructures of Ti80 alloy subjected to three heat treatments are shown in Figure 5. The low-magnification SEM image indicates that all specimens were dominated by the equiaxed α_p_ phase and β_t_ phase, and the distribution of equiaxed α_p_ was uniform (Figure 5a–c). The microstructure characteristics of equiaxed α_p_ are listed in Table 2. The results show that the volume fraction and the average width of equiaxed α_p_ phase had no significant differences among the three samples, which indicates that aging treatment at 600 °C had a minimal effect on the equiaxed α_p_ morphology. However, the acicular phase can be observed in the magnified SEM results (Figure 5(a1–c1)). Further TEM characterization was conducted to identify the acicular phase in β_t_. As can be seen in Figure 5(a2), the needle-like precipitated phase in β_t_ of the ST specimen was very fine, with an average thickness of ~260 nm. After subsequent aging, the acicular phase in β_t_ grew considerably larger, as shown in Figure 5(b2,c2). With the increase in the aging time from 5 h to 24 h, the thickness of the acicular phase from ~430 nm (STA-1) increased to ~690 nm (STA-2). The acicular phase can be identified from the selected area electron diffraction (SAED) as the HCP structure in all samples. During rapid cooling from a high temperature, β transformed into α’ martensite through displacive transformation in near-α titanium alloys. This caused the elemental composition of the α’ phase to be identical to that of the parent β phase [9,24,25]. During the subsequent aging process, α’ decomposed into α_s_+β. This is a diffusive phase transformation. Therefore, there are elemental differences between α’ and α_s_. Specifically, α-stable elements are enriched in α_s_, while β-stable elements are enriched in α’ [26]. Based on the aforementioned studies, it is speculated that the acicular phase in the β_t_ phase of heat-treated samples can be either α_s_ or α’. Consequently, HAADF-STEM was used to identify them.

**Figure 6:**
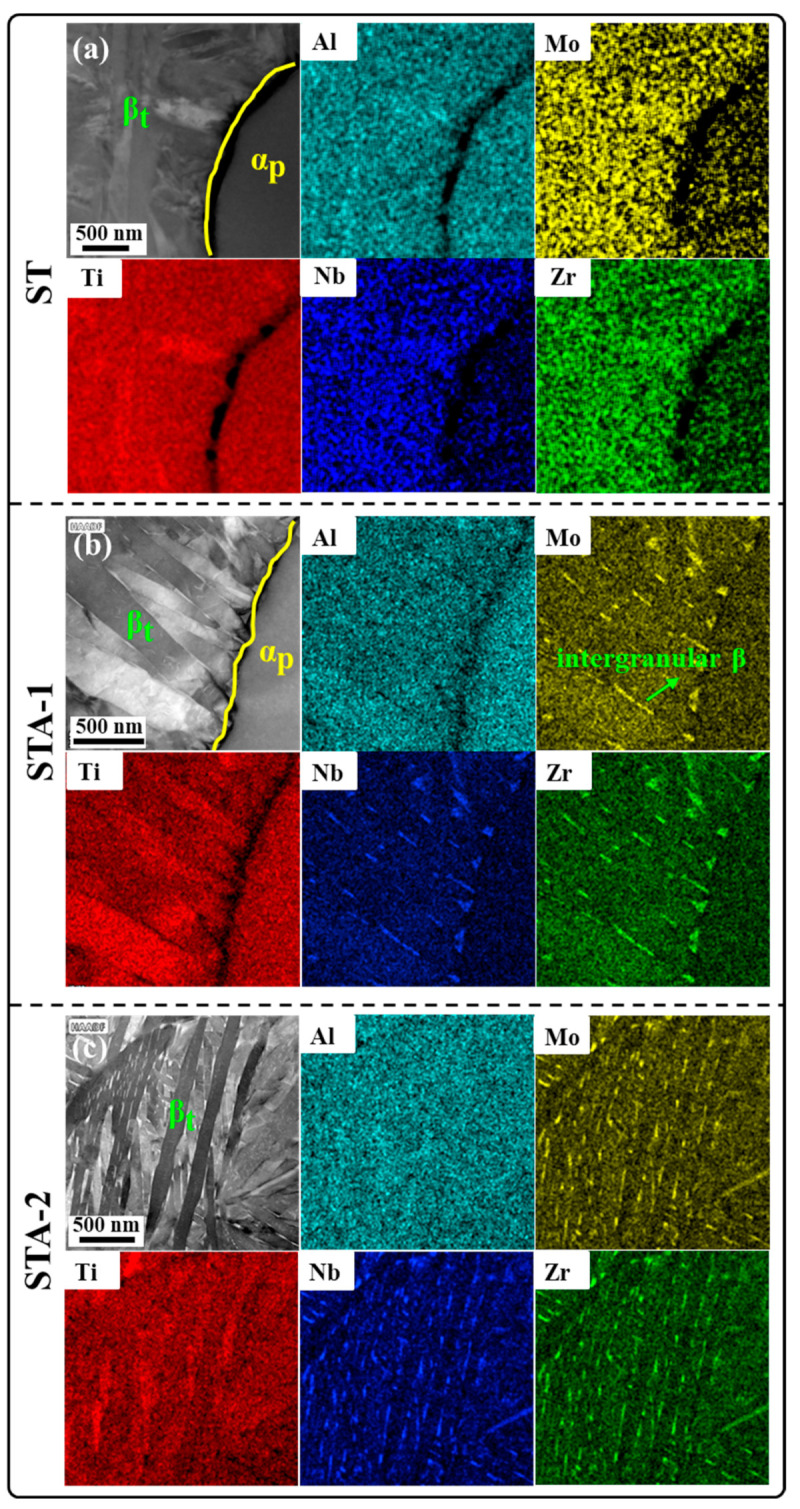
HAADF-STEM images and corresponding element EDS mapping results for (**a**) ST; (**b**) STA-1; (**c**) STA-2.

Figure 6 presents high-angle annular dark-field scanning transmission electron microscopy (HAADF-STEM) images and the corresponding elemental energy-dispersive X-ray spectroscopy (EDS) results of the Ti80 alloy following three heat treatments. Al is an α-stable element which is enriched in the α phase. Nb and Mo are β-stable elements which are enriched in the β phase and the α’ phase. Zr is a neutral element [26]. However, it has been demonstrated by various studies that Zr is an effective alloying element in stabilizing the β phase at room temperature. The addition of Zr resulted in a reduction in martensitic transformation temperatures, which indicates that Zr can be employed as a β-stable element [27,28]. As for the ST specimen, the contents of Mo, Nb, and Zr in β_t_ were significantly higher than those in equiaxed α_p_, but there was no enrichment in the β_t_ phase, as can be seen from Figure 6a. Therefore, there was no element enrichment between the acicular phase and the matrix in β_t_, suggesting that the lamellar phase in β_t_ of the ST specimen was α’ phase. After subsequent aging treatment, the enrichment degrees of Mo, Nb, and Zr in β_t_ were significantly different (Figure 6b,c), which suggests that the lamellar α in β_t_ of STA-1 and STA-2 samples was α_s_. Meanwhile, Mo, Nb, and Zr were discontinuously enriched at the lamellar α grain boundaries, which formed an intergranular β phase (Figure 6b,c).

**Figure 7:**
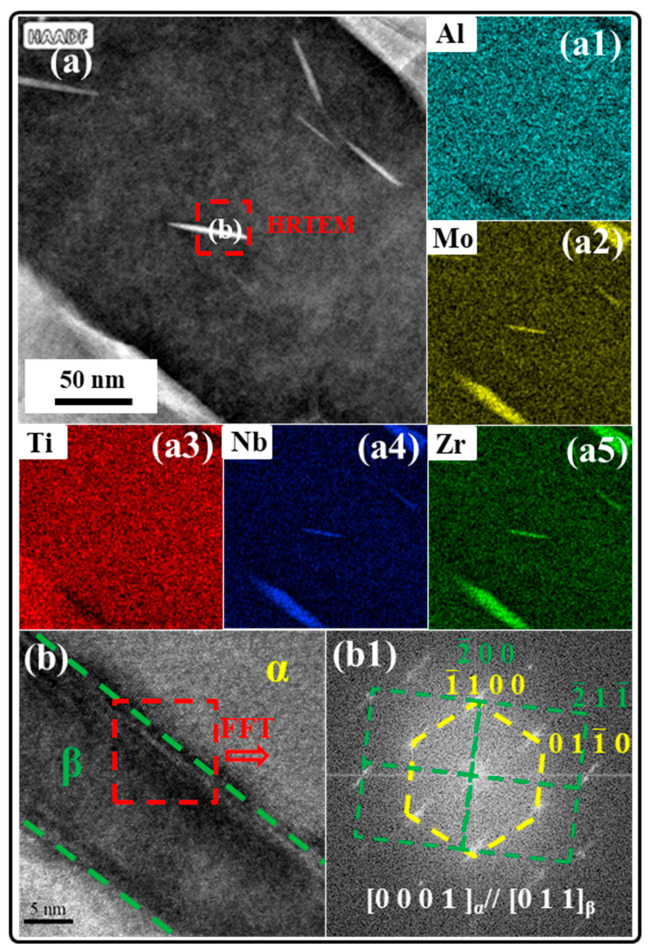
(**a**–**a5**) HAADF-STEM images of STA-1 sample and the corresponding elemental EDS mapping results; (**b**,**b1**) HRTEM image corresponds to the red dotted rectangle in (**a**).

Meanwhile, for the STA-1 sample, an interesting phenomenon was observed at HAADF-STEM, that is, the presence of a nano-precipitated phase within the lamellar α, as shown in Figure 7a. In order to further determine the composition and microstructure of the nano-precipitated phase, HAADF-STEM mapping and high-resolution TEM (HRTEM) were used, and the results are shown in Figure 7(a1–a5) and Figure 7(b,b1), respectively. The results of HAADF-STEM mapping indicate that the elements of Nb and Mo were enriched on the nano-precipitated phase. The results of fast Fourier transform (FFT) of Figure 7(b1) indicate that the matrix phase had an HCP structure, and the nano-precipitated phase had a BCC structure. Furthermore, the nano-precipitated phase maintained BOR with the matrix, i.e., (0001)α∥(011)β, <11$\bar{2}$0>α∥<111>β [29]. This shows that the matrix phase is the α phase and the nano-precipitated phase is the intragranular β phase. It can be indicated that the nano-β is derived from the decomposition of α’; thus, the alloy undergoes an α’ → α+β decomposition reaction during aging at 600 °C [30,31].

**Figure 8:**
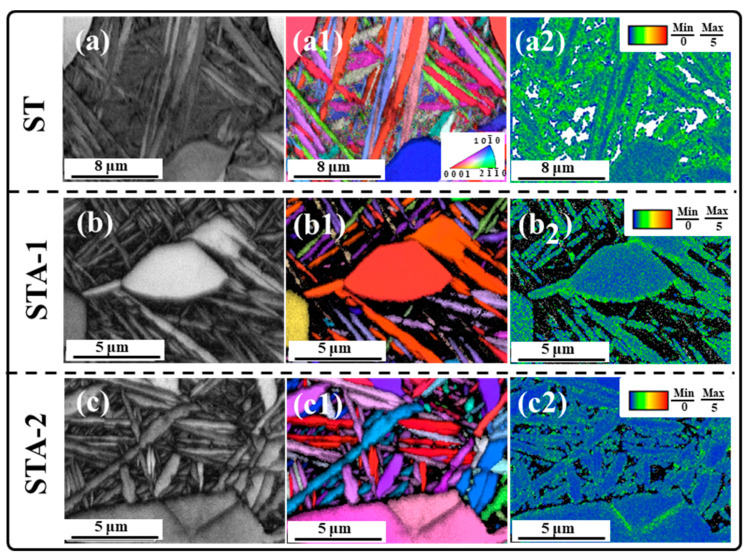
EBSD maps of the alloy after heat treatment. (**a**–**c**) IQ mappings; (**a1**–**c1**) IPF mappings; (**a2**–**c2**) KAM mappings.

As shown in the TEM images in Figure 5(a3–c3), dislocations could be observed in the three heat-treated specimens. To further investigate the differences in dislocation density in these samples, EBSD characterization was carried out. Figure 8 shows the EBSD results of the Ti80 alloy subjected to three heat treatments, including image quality mappings (IQ), inverse pole figure mappings (IPF), and Kernel average misorientation mappings (KAM). The KAM value qualitatively indicates the density of dislocation [32,33]. It can be seen from Figure 8(a2) that high KAM values were present in the ST sample, and the KAM values of equiaxed α_p_ were lower than those of β_t_. This indicates that a considerable number of dislocations were generated at β_t_ in order to accommodate the strain of β→α’. After subsequent aging treatment, the KAM values of the STA-1 and STA-2 samples decreased significantly, indicating that the dislocation density decreased dramatically (Figure 8(b2,c2)).

## 4. Discussion

### 4.1. Effect of α’ Decomposition on Microstructure Evolution

**Figure 9:**
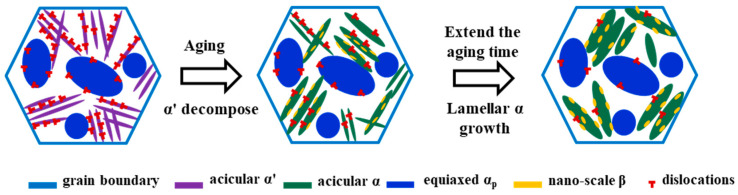
Schematic diagram of the microstructure evolution of Ti80 alloy during solution treatment and aging.

Figure 9 schematically shows the microstructure evolution of the Ti80 alloy during solution treatment in the α+β phase region and aging. Due to the rapid cooling during solution treatment in the α+β phase region, the Ti80 alloy deviated from the equilibrium state. Meanwhile, a significant number of dislocations were generated within the alloy in order to accommodate the strain produced by the martensitic phase transition during cooling. Thus, the Gibbs free energy of the alloy increased. At this stage, the microstructure of the Ti80 alloy consisted of equiaxed α_p_ and β_t_ (acicular α’+residual β). Large amounts of Nb and Mo atoms were enriched in the acicular α’ phase. Subsequent aging led to a decrease in the Gibbs free energy, resulting in a decrease in dislocations and the decomposition of the α’ phase into α_s_ and nano-β (intergranular β, intragranular β). The thermodynamic origin of the decomposition of the α’ phase in Ti80 is the decrease in Gibbs free energy [34]. Furthermore, the decomposition of α’ can also be attributed to aging at a high temperature and concentration gradients of elements such as Nb, Mo, and Zr atoms. The aging process caused interdiffusion of β-stable and α-stable elements, resulting in the formation of the lamellar α_s_ phase and the nano-β phase. This process led to the enrichment of Mo and Nb atoms in the nano-β. In conclusion, this decomposition process is an element-controlled diffusion process which also involves a reduction in dislocation density [35]. Meanwhile, with prolonged aging time, the sizes of the of α_s_ and nano-β phases gradually increase, and the dislocation density decreases. It is of interest to note that, due to the fixed solution treatment temperature, the morphology of equiaxed α_p_ does not undergo significant changes during solution treatment and aging.

### 4.2. Effect of Microstructure Evolution on Mechanical Properties

The strengthening mechanisms of titanium alloys can be broadly categorized into: solution strengthening, second-phase strengthening, α-phase lattice strengthening, fine-grain strengthening, texture strengthening, etc. [36,37]. As mentioned above, in this study, the factors that led to changes in the mechanical properties of the alloy mainly include the initial dislocation density, the size effect of α_s_, and nano-β.

**Figure 10:**
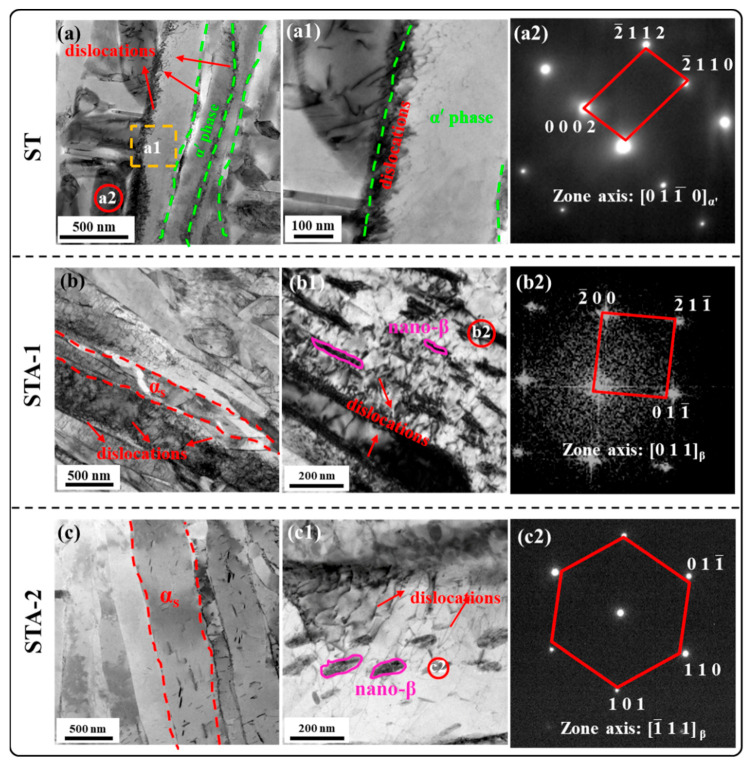
TEM images of heat-treated Ti80 alloy after tensile test near fracture. (**a**–**c**) Low magnification BF image; (**a1**–**c1**) High magnification BF image; (**a2**–**c2**) SAED pattern corresponding to the red circle in (**a**,**b1**,**c1**).

For the ST specimen, it can be seen from the KAM mapping in Figure 8(a3) that, due to the lattice distortion caused by the martensitic phase transformation, a high density of dislocations was generated in the β_t_; hence, the ST sample exhibited good strength, with 1083 MPa. However, by comparing Figure 8(a3,b3), it was found that the KAM value of the STA-1 specimen was lower than that of ST, but the strength of the STA-1 specimen (1126 MPa) was higher than that of the ST sample (1083 MPa). Thus, the decrease in dislocation density was not a factor in the increase in the strength of the STA-1 sample. Hence, in order to further reveal the differences in the failure mechanisms of the samples, after the tensile test, the specimens were subjected to TEM characterization, and the results are shown in Figure 10.

As can be seen from Figure 10(a,a1), after the tensile test, significant dislocation plugging and entanglement could be observed at the α’ interface in the ST specimen, while the dislocation density was low inside the α’. For the STA-1 sample, after the tensile test, a large number of dislocations was observed not only at the α_s_ interface, but also entangled around the nano-β phase inside the α_s_, as shown in Figure 10(b,b1). In summary, the reason for the increase in the strength of the STA-1 sample was that, during the aging process, the α’ martensite decomposed to the α_s_ and nano-β phases; meanwhile, the α_s_ and nano-β phase could effectively hinder the dislocation motion during the deformation of the alloy [38].

Compared to ST and STA-1 specimens, the strength of the STA-2 specimen decreased significantly. For the STA-2 specimen, it can be seen from the KAM mapping in Figure 8(c3) that, with the aging time extended to 24 h, the dislocation density in the alloy was significantly lower than that of ST and STA-1, which is one of the reasons for the decline in the strength of the STA-2 specimen. In addition, as can be seen from Figure 10(c,c1), a number of entangled dislocations was observed around the nano-β phase, and this result is similar to that of the STA-1 sample. However, for the STA-1 and STA-2 specimens, there was an obvious difference in the width of the lamellar α_s_ and nano-β phase. With the aging time extended from 5 h to 24 h, the α_s_ lamella thickness increased from ~430 nm in the STA-1 specimen to ~688 nm in the STA-2 specimen. Therefore, the increase in α_s_ and nano-β phase thickness was another factor that decreased the strength of the STA-2 sample.

In accordance with the aforementioned principle, the yield stress of the Ti80 alloy can be expressed as follows [39]:

σ = σ_0_ + ψ_σp_σ_p_ + ψ_σs_σ_s_ + ψ_β_σ_β_ (1)

where σ_0_ stands for the natural lattice friction; σ_p_ denotes the strength of the equiaxed α_p_; σ_s_ represents the strength of the lamellar αs/α’; σ_β_ is the strength of the precipitated phase β; and ψ_σp_, ψ_σ_, and ψ_β_ denote the volume fractions of the α_p_, σ_s_, and precipitated phase β phases, respectively.

In accordance with the classical Hall–Petch relationship, σ_p_ is related to the average size of α_p_ grains [40]:

(2) $$\sigma_{p}\;=\;\sigma_{0}+k_{\alpha_{p}}d_{\alpha_{p}}^{-\frac{1}{2}}$$

Herein, σ_0_ and $k_{\alpha_{p}}$, are the frictional stress and Hall–Petch coefficient of the α_p_, phase, respectively, and $d_{\alpha_{p}}$ is the average diameter of the α_p_ phase. During the solution treatment and aging, the morphology and size of the equiaxed α_p_ phase do not change significantly. Thus, in this study, the change in mechanical properties was not dependent on the α_p_ phase.

The semi-empirical Hall–Petch relation indicates that the relation of σ_s_ can be expressed as follows [41]:

σ_s_ = σ_0_ + k(w_αs/α’_/2)^−1/2^ (3)

where σ_0_ and k are the friction stress of α_s_/α’ and the Hall–Petch coefficient, respectively, and w_αs/α’_ is the width of the α_s_/α’ phase. The α_s_/α’ phase has been demonstrated to enhance the strength of titanium alloys, with the observed increase in strength correlating with a reduction in the width of the α_s_/α’ phase [42,43]. Prolonging the aging time, the width of the α_s_ phase increases, and the effect of the layer thickness on the alloy’s mechanical properties is more significant.

The σ_β_ can be expressed by the following formula:

σ_β_ = σ_0_ + k_β_d_β_^−1/2^ (4)

where σ_0_ and k_β_ are the frictional stress and Hall–Petch coefficient of the precipitated β phase, respectively. d_β_ represents the average diameter of the nano-β phase. The equation demonstrates that as the aging time is extended to 24 h, the volume fraction of the precipitated phase increases, yet the strength of the alloy decreases due to the nano-β size effect.

**Figure 11:**
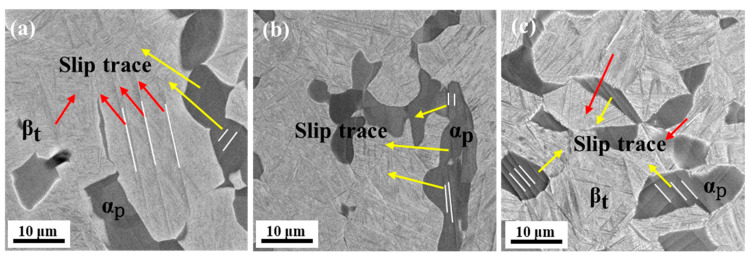
SEM images of different heat-treated samples of Ti80 alloy at a strain of 3%, where red arrows represent slip traces in β_t_ and yellow arrows in α_p_. (**a**) ST; (**b**) STA-1; (**c**) STA-2.

Considering that there is a difference in α_p_ and β_t_ plastic deformation capacity in titanium alloys, in order to further reveal the roles assumed by β_t_, which contains α_s_ and α’, in the plastic deformation process of the three samples, hardness tests and tensile experiments with strains up to 3% were conducted—3% indicates that the strains of ST, STA-1, and STA-2 samples are just passing the yield point. At this time, the softer phase in the alloy preferentially undergoes plastic deformation, resulting in the formation of a slip trace [44,45]. Table 3 shows that the hardnesses of β_t_ in the ST, STA-1, and STA-2 samples were 331 HV, 354 HV, and 301 HV, respectively. The hardness of β_t_ increased and then decreased. The microstructures of specimens with tensile strains up to 3% are shown in Figure 11. For the ST sample, some slip traces were observed in both the β_t_ and α_p_ phases, as is presented in Figure 11a. For the STA-1 specimen, as shown in Figure 11b, it can be observed that the slip traces only appeared within the equiaxial α_p_ phase. This situation indicates that, due to α’ decomposition, the hardness of the β_t_ phase increased, resulting in plastic deformation occurring preferentially within the soft α_p_ phase, which is consistent with the results regarding the hardness values of β_t_. The experimental results for the STA-2 sample are very different from those of the ST and STA-1 samples. A large number of slip traces were observed in both the α_p_ and β_t_ phases, as can be seen in Figure 11c. The reason for this phenomenon was that, after the 24 h aging treatment, the increase in the size of the α_s_ phase and nano-β, as well as the significant decrease in dislocation density, led to a significant decrease in the hardness of the β_t_ matrix. As a result, the deformation compatibility of STA-2 specimen was dramatically improved, favoring the increase in alloy ductility. The study suggests that the α’ decomposition, which leads to an increase in β_t_ hardness, was the primary reason for the significant increase in strength in the STA-1 sample.

## 5. Conclusions

In this study, the microstructure characteristics and mechanical properties of Ti80 alloy subjected to solution treatment within the α+β phase region and aging were comparatively investigated. The present work aimed to explore the decomposition mechanisms of the α′ phase in aging, as well as its influence on the microstructure and mechanical properties. The main conclusions can be summarized as follows:

(1) The YTS, UTS, and elongation of the ST sample reached 898 MPa, 1083 MPa, and 7.7%, respectively. Then, after subsequent aging for 5 h, the strength and plasticity of the STA-1 specimen were synergistically improved, with the YTS, UTS, and elongation reaching 1012 MPa, 1126 Mpa, and 8.1%, respectively. With further extension of the aging time to 24 h, the YTS and UTS of the STA-2 sample were reduced to 871 MPa and 958 Mpa, respectively, and the elongation increased to 10.2%.
(2) The microstructures of the three specimens were mainly composed of equiaxed α_p_ and α_s_ (α’). For ST sample, the microstructure consisted of equiaxed α_p_ + acicular α’. After subsequent aging, α’ decomposed into α_s_ and nano-β phases in STA-1 and STA-2 specimens. Furthermore, as the aging time was extended, the dislocation density decreased, while the size of the α_s_ and nano-β phases gradually increased.
(3) The mechanical properties of the heat-treated samples were affected by the initial dislocation density, the size effect of α_s_, and nano-β. The fine lamellar α_s_ and nano-β phase can effectively impede the movement of dislocations during plastic deformation, thus achieving alloy strengthening. Based on the above principles, a microstructures–properties correlation model of Ti80 alloy was constructed.
(4) The distribution of slip traces in the supplementary 3% tensile experiments of heat-treated specimens was different. This suggests that α’ decomposition results in an increase in β_t_ hardness, which was the primary cause of the significant strength increase observed in the STA-1 specimen.

## Figures and Tables

**Table 1:** Chemical composition of Ti-6Al-3Nb-2Zr-1Mo alloy (wt.%).

Element	Al	Nb	Zr	Mo	Si	Fe	O	C	N	H	Ti
Wt.%	6.30	3.04	2.04	1.07	<0.03	0.1	0.039	0.0085	0.0040	<0.001	Bal.

**Table 2:** Volume fraction of the α_p_ phase and the widths of the α_p_ phases for the samples.

Heat Treatment Samples	Fraction of α_p_ Phase/%	Width of α_p_ Phase/μm
ST	31.1 *±* 3.7	8.0 *±* 0.3
STA-1	33.5 *±* 1.2	7.9 *±* 0.4
STA-2	32.0 *±* 3.0	8.1 *±* 0.3

**Table 3:** Vickers hardness for the samples under different heat treatment processes.

Sample	Vickers-Hardness/HV
ST	331 *±* 4
STA-1	354 *±* 7
STA-2	301 *±* 5

## Data Availability

The original contributions presented in the study are included in the article material, further inquiries can be directed to the corresponding author.
